# Glial peroxisome dysfunction induces axonal swelling and neuroinflammation in *Drosophila*

**DOI:** 10.1093/g3journal/jkae243

**Published:** 2024-10-10

**Authors:** Maggie Sodders, Anurag Das, Hua Bai

**Affiliations:** Department of Genetics, Development, and Cell Biology, Iowa State University, Ames, IA 50011, USA; Department of Genetics, Development, and Cell Biology, Iowa State University, Ames, IA 50011, USA; Department of Genetics, Development, and Cell Biology, Iowa State University, Ames, IA 50011, USA

**Keywords:** peroxisomal import, glia-axon communication, wrapping glia, Pex5, Gnpat, Acox1, JAK-STAT

## Abstract

Glial cells are known to influence neuronal functions through glia–neuron communication. The present study aims to elucidate the mechanism behind peroxisome-mediated glia–neuron communication using *Drosophila* neuromuscular junction (NMJ) as a model system. We observe a high abundance of peroxisomes in the abdominal NMJ of adult *Drosophila*. Interestingly, glia-specific knockdown of peroxisome import receptor protein, *Pex5*, significantly increases axonal area and volume and leads to axon swelling. The enlarged axonal structure is likely deleterious, as the flies with glia-specific knockdown of *Pex5* exhibit age-dependent locomotion defects. In addition, impaired peroxisomal ether lipid biosynthesis in glial cells also induces axon swelling. Consistent with our previous work, defective peroxisomal import function upregulates pro-inflammatory cytokine upd3 in glial cells, while glia-specific overexpression of *upd3* induces axonal swelling. Furthermore, motor neuron-specific activation of the JAK-STAT pathway through *hop* overexpression results in axon swelling. Our findings demonstrated that impairment of glial peroxisomes alters axonal morphology, neuroinflammation, and motor neuron function.

## Introduction

Peroxisomes play crucial roles in regulating cellular redox homeostasis, oxidation of very long-chain fatty acids (VLCFAs), and biosynthesis of ether phospholipid (e.g. plasmalogen) (Wanders and Waterham 2006; Lodhi and Semenkovich 2014). Since most of the peroxisomal matrix proteins are synthesized in the cytosol (like catalase and peroxisomal acyl-CoA oxidase), all peroxisomal functions are dependent on the import of matrix proteins into the organelle, which is controlled by a set of unique peroxisomal biogenesis proteins named peroxins (PEXs) in both plants and animals (Platta and Erdmann 2007; Ma *et al*. 2011; Hu *et al*. 2012; Meinecke *et al*. 2016; Francisco *et al*. 2017). Pex5 is the primary receptor protein involved in peroxisomal protein import, which typically binds to matrix proteins with a C-terminal peroxisome targeting sequence 1 (PTS1) (Gould *et al*. 1989; Stanley *et al*. 2006). In humans, mutations of PEX family genes cause disrupted peroxisome activity, leading to peroxisome biogenesis disorders (PBDs), including Zellweger syndrome (Zalckvar and Schuldiner 2022; Wanders *et al*. 2023). Recently, peroxisomal dysfunction has emerged as an essential contributing factor to prevalent human diseases, including diabetes, cancer, and neurodegenerative diseases (Zalckvar and Schuldiner 2022).

Mutations in PEXs or peroxisomal matrix enzymes cause a range of symptoms affecting several organ types and systems, including the liver, renal, and central nervous system (CNS). Neuronal symptoms of these disorders can include neurodevelopmental delays and neurodegeneration (Wanders *et al*. 2023), although the underlying mechanisms are still unclear. PBD patients often develop severe neurological abnormalities, including axonal demyelination and degeneration, neuronal migration defects, and neuroinflammation (Berger *et al*. 2016). Despite the cell-autonomous function of peroxisomes within neurons, we know very little about the role of glial peroxisomes in neuroprotection and how peroxisomes contribute to neuron–glia communication. To date, only a few previous studies have attempted to experimentally test the impact of glial peroxisome deficiency on neuronal function and integrity. *Drosophila* larvae with *Pex1* mutations exhibit abnormal neuronal and glial development (Mast *et al*. 2011). Oligodendrocyte-specific *Pex5* knockout (KO) mice exhibit a range of neuronal and neuromuscular phenotypes, as well as elevated neuroinflammation (Kassmann *et al*. 2007). Glial tissue, however, seems largely unaffected by *Pex5* KO. In contrast, mice with peroxisome-deficient oligodendrocytes suffered axonal loss and reduced motor function (Kassmann *et al*. 2007). In *Drosophila*, acyl-CoA oxidase 1 (*dACOX1*) null mutants exhibit severe neurodegeneration of the retina and progressive vision loss, which can be rescued by ectopic expression of *UAS-human ACOX1* (Chung *et al*. 2020). Interestingly, loss of *ACOX1* causes glial degeneration, which is likely due to the accumulation of VLCFAs and elevated reactive oxygen species (ROS) production (Chung *et al*. 2020). However, it remains unclear how glia–neuron communication is regulated under peroxisome dysfunction, and the intertissue communication factors have not been identified.

Similar to the vertebrate system, *Drosophila* glial cells play crucial roles in neuronal protection and homeostatic regulation. Various glial subtypes have been previously identified in different developmental stages and are spatially distributed in different regions of the nervous system, including cortex glia in the cortical areas of the CNS, ensheathing and astrocyte-like glia in the neuropile, and wrapping glia in the peripheral nervous system (PNS) (Awasaki *et al*. 2008; Freeman 2015; Kremer *et al*. 2017). In the PNS, 3 glial subtypes, wrapping glia, subperineural glia, and perineural glia, ensheath the axon of the motor neurons and support the function of motor neurons and other peripheral sensory neurons (Hartenstein 2011). In *Drosophila* neuromuscular junction (NMJ), wrapping glia ensheathment of the axons of motor neurons has been phenotypically linked to Remak bundles, the unmyelinated axon bundles in the mammalian system (Stork *et al*. 2008). In addition, subperineural glia extend further and interact with the synaptic regions of the NMJ (Freeman 2015).

In the present study, we aim to use *Drosophila* NMJ as a model system to dissect the role of glial peroxisome in neuronal function and to uncover the secretory factors mediating glia–neuron communication. We also examine the potential involvement of pro-inflammatory cytokine in glia–neuron communication and peroxisome-regulated neuroinflammation. Our findings demonstrated that glia-specific knockdown (KD) of *Pex5* significantly increases axonal area and volume in adult *Drosophila* NMJ, which is associated with age-dependent locomotion defects. KD of *Pex5* upregulates pro-inflammatory cytokine upd3 in glial cells, while either glia-specific overexpression of *upd3* or motor neuron-specific activation of JAK-STAT causes axonal swelling. Taken together, our findings suggest that glial peroxisomal defects impact axonal morphology and motor neuron function by upregulating JAK-STAT signaling and neuroinflammation.

## Material and methods

### Fly husbandry and stocks

Flies were maintained at 25°C, 40% relative humidity and 12-h light/dark. Adults were reared on agar-based diet with 0.8% cornmeal, 10% sugar, and 2.5% yeast (unless otherwise noted). Fly stocks used in the present study are *Mz97-GAL4* and *Moody-GAL4* (gifts from Elizabeth McNeill), *repo-GAL4* (a gift from Elizabeth McNeill), *UAS-upd3* (a gift from Doug Harrison), *UAS-hop*^*Tuml*^ (a gift from Erika Bach), *upd3-lacZ* (a gift from Bruce Edgar), *Repo-GS-GAL4* (a gift from Amita Sehgal), *OK6-GAL4* (RRID:BDSC_64199), *UAS-Pex5 RNAi* (RRID:BDSC_58064), *UAS-Acox1 RNAi* (RRID:BDSC_52882), *UAS-Gnpat* RNAi (RRID:BDSC_52914), and *UAS-Catalase* RNAi (RRID:BDSC_31894). Several control lines were used in the study: *y1 v1; P[CaryP]attP40 (*RRID:BDSC_36304), *w^1118^* (RRID:BDSC_5905), and *yw^R^* (a gift from Marc Tatar). Adult female flies were used in this study, as females are larger than males and are easy to dissect. For most experiments, adults are raised to 7 days posteclosion, unless indicated otherwise. RU486 (mifepristone, Sigma, St. Louis, MO, USA) was used to activate *Repo-GS-GAL4* at a final concentration of 200 μM mixed into the flies’ food. The FlyBase was used for *Drosophila* gene annotation (Öztürk-Çolak *et al*. 2024).

### Adult abdominal dissection

Adult *Drosophila* were anesthetized using flynap (Carolina, Burlington, NC, USA) and placed in a dish with a light layer of Vaseline coating the bottom. Using scissors, a cut was made between the thorax and the abdomen. Flipped carcasses abdomen were incubated with Ca^2+^-free media (128 mM NaCl, 2 mM KCl, 4 mM MgCl2(H2O)6, 35.5 mM sucrose, 5 mM HEPES, 1 mM EGTA, H2O, pH 7.2). Internal organs were removed from the carcass to expose abdominal ventral longitudinal muscle (VLM), as described in our previous studies (Birnbaum *et al*. 2020).

### Immunostaining

Tissues were fixed with 4% paraformaldehyde for 20 min at room temperature, followed by washing in 1× PBST (0.1% Triton X) and blocking with 5% normal goat serum. Tissues were then incubated with primary antibodies overnight at 4°C, with secondary antibodies for 1 h at room temperature. Samples were washed in 1× PBST and mounted in Prolong Diamond (Life Technologies). Primary antibodies used were as follows: anti-repo (DSHB #8D12), anti-ATP5A1 (Fisher #439800), anti-Beta-galactosidase (Promega #Z3781), anti-HRP-Alexa Fluor 594 (Jackson ImmunoResearch Laboratories Inc.), and anti-Fly PMP70 (a gift from Kyu-Sun Lee Lab). The primary antibody dilution was 1:200. Tissues were then washed in 1× PBST. Tissues were then incubated for 1.5 h at room temperature in 1× PBST at a dilution of 1:500 with secondary antibodies and kept in the dark at room temperature. Secondary antibodies (1:500 dilution of 1.5 mg/ml stock solution) were as follows: Alexa Fluor 488 anti-mouse, Alexa Fluor 647 anti-mouse, and Alexa Fluor 488 anti-guinea pig (Jackson ImmunoResearch Laboratories Inc., West Grove PA).

### Imaging and analysis

Images were captured using Olympus FV3000 laser scanning confocal (Olympus, Waltham, MA, USA). Using a 40× lens with 20× digital zoom, images were taken from axon 1 node above the NMJ terminal. Analysis was performed on ImageJ/Fiji. The entire axon is contained in the *Z*-slices of the images taken. For area analysis, the max projection of the images was analyzed using the polygon tool in ImageJ to trace the outline of axon. For volume analysis, image stacks were first processed using OTSU threshold. The volume of the axon was calculated using the standard formula for cylindrical volume on ImageJ: volume = pi ∗ radius2 ∗ height.

### Climbing assay

Climbing ability was measured via a negative geotaxis assay performed by tapping flies to the bottom of an empty glass vial and counting flies that climbed at different positions of the vial. The percentage of flies was counted in each of the 3 sections (0–3, 3–6, and 6–9 cm), 10 s after being tapped down. The climbing ability was calculated by comparing the percentage of flies in the top-most portion vs the middle portion and the bottom portion. About 5–10 vials (10 females per vial) were tested per group.

### RNA extraction and quantitative RT-PCR

To validate RNAi KD efficiency (Supplementary Fig. 1), 5–10 female flies with global KD of target genes (using *da-GAL4*, a gift from Marc Tatar) were homogenized using TissueLyser II (QIAGEN), and RNA was extracted using TRIzol reagent (Thermo Fisher Scientific). DNase-treated total RNA was then reverse transcribed to cDNA using iScript cDNA Synthesis Kit (Bio-Rad). Quantitative RT-PCR (qRT-PCR) was performed with a Quantstudio 3 Real-Time PCR system and PowerUp SYBR Green Master Mix (Thermo Fisher Scientific). Three independent biological replicates were performed. The mRNA abundance of each candidate gene was normalized to the expression of *RpL32* by the comparative CT methods. Primer sequences are listed in the following: *RpL32*: forward 5′-AAGAAGCGCACCAAGCACTTCATC-3′ and reverse 5′-TCTGTTGTCGATACCCTTGGGCTT-3′; *Pex5*: forward 5′-GATGTGGAGAACCCGTTTGA-3′ and reverse 5′-CGCCACCTCGAAACATAGAA-3′; *Acox1*: forward 5′-CGTGCTTACCTACGGAACTATG-3′ and reverse 5′-TCGGCGAACAGCTGAATAC-3′; *Gnpat*: forward 5′-GGAGGTGGAACTAGTCAAACAG-3′ and reverse 5′-GACACTCCTTTGGGCATACA-3′; and *Cat*: forward 5′-GGTTTCTCCTGGTGCCATTA-3′ and reverse 5′-GAAGTGCGACATCTCATCCA-3′.

### Statistics

GraphPad Prism (GraphPad Software) was used for statistical analysis. Unpaired 2-tailed Student's *t*-test or 1-way ANOVA (Tukey multiple comparison) was performed to compare the mean value between control and treatment groups. The outliers were excluded using Robust regression and Outlier removal (ROUT) method (*Q* = 1%) prior to the data analysis.

## Results

### Glia-specific KD of *Pex5* causes axonal swelling of peripheral motor neurons and impairs locomotion activities

**Fig. 1.**
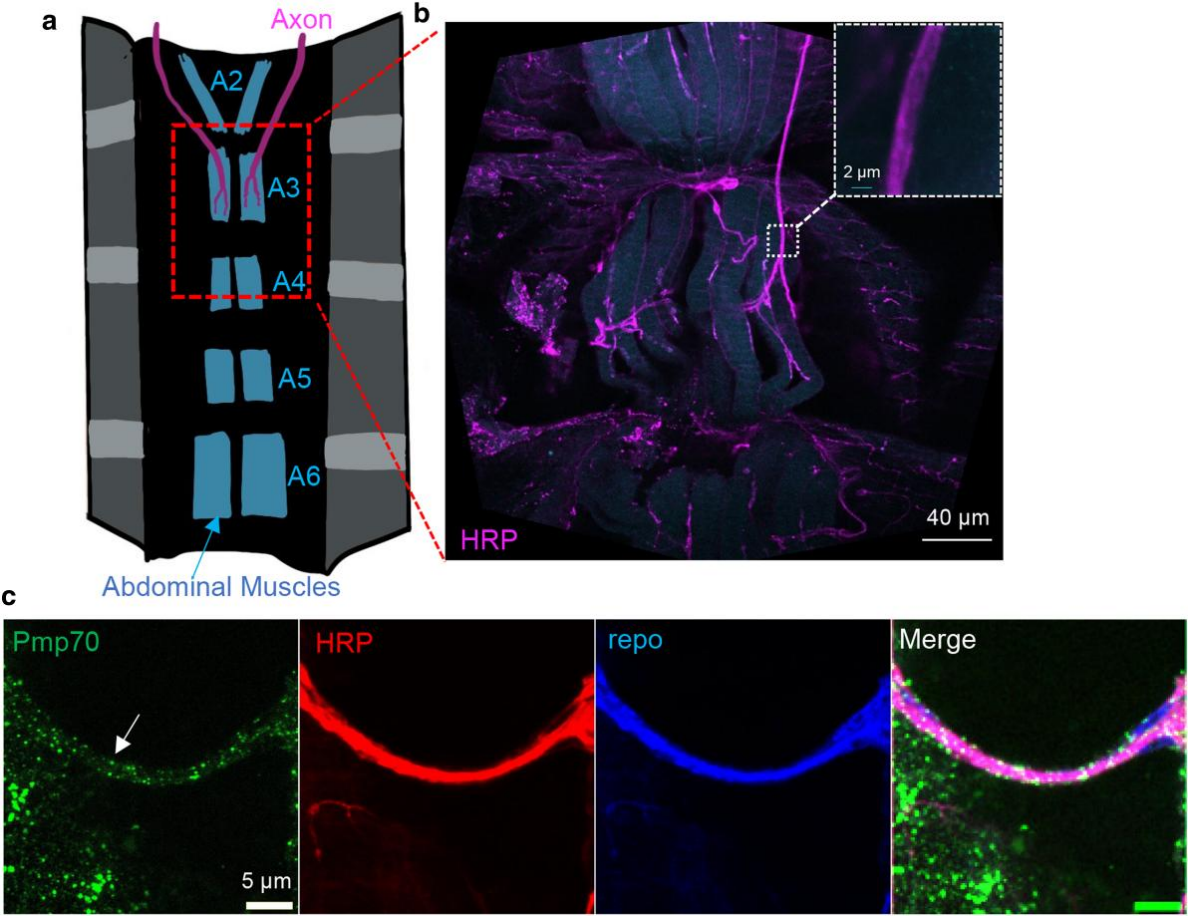
Peroxisomes are enriched in the glial and neuronal tissues of the abdominal VLM in adult *Drosophila*. a) Schematic diagram showing adult *Drosophila* abdominal muscles and axon innervation of motor neurons. b) Representative image showing neuronal tissues of the A3 abdominal VLM that are labeled with anti-horseradish peroxidase (HRP) antibodies. The dashed box indicates the area of interest used for axonal area and volume quantifications. c) Representative image showing the presence of peroxisomes in the axon of motor neurons. Peroxisomes are labeled with anti-Pmp70 antibodies (white arrow). Glia are labeled with anti-repo antibodies, while neuronal tissues are labeled with anti-HRP antibodies.

**Fig. 2.**
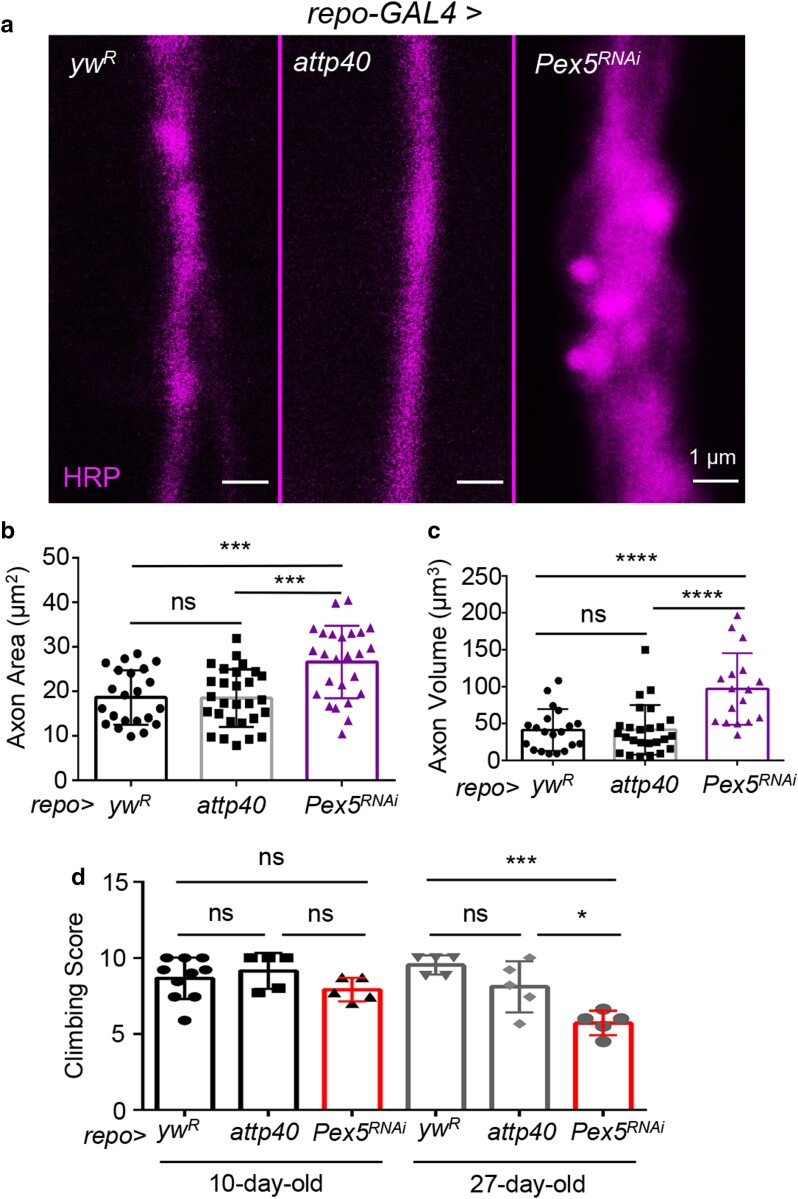
Glia-specific KD of *Pex5* causes axonal swelling of peripheral motor neurons and impairs locomotion activities. a) Representative images showing axon morphology in 2 wild-type flies (the left and middle panels) and glia-specific *Pex5 RNAi* flies (the right panel). Two control crosses are shown, *repo-GAL4 > yw^R^* and *repo-GAL4 > P(CaryP)attp40*. Axons are labeled with anti-HRP antibodies. b, c) Quantification of axonal area and volume in flies with glia-specific *Pex5* KD. *N* = 20, 1-way ANOVA. d) Climbing activity of glia-specific *Pex5* KD flies at 2 different ages. About 5–10 vials (10 females per vial) were tested per group. *N* = 5–10, 1-way ANOVA. *P < 0.05, **P < 0.01, **P < 0.001, and ****P < 0.0001.

To study the role of peroxisome in glia–neuron communication, we focus on the adult NMJ at the A3 segment of the abdominal VLM (Fig. 1a and b). Our previous work demonstrated that the abdominal VLM is a great model for aging and neuronal homeostasis studies (Birnbaum *et al*. 2020). Peroxisomes, as labeled by antibodies against Pmp70, were confirmed to be present in the glial and neuronal tissues in the A3 abdominal VLM region (Fig. 1c). Using a pan-glial driver (*repo-GAL4*) (Awasaki *et al*. 2008; Doherty *et al*. 2009), we knocked down *Pex5* specifically in glial cells and examined the changes in axonal structure at 7 days of age. Similar to the previous studies in oligodendrocyte-specific *Pex5* KO mice (Kassmann *et al*. 2007), we found that adult *Drosophila* with impaired peroxisomal function in glial cells showed significantly enlarged axons, both axonal area and volume (Fig. 2a–c). Two control crosses were performed, and both confirmed the effects of *Pex5* KD. These findings suggest that glial peroxisomes are essential in maintaining axonal integrity, and loss of glial peroxisome function promotes axonal swelling, which is likely through the induction of neuroinflammation.

A negative geotaxis assay was performed to further examine the impact of axonal swelling on motor neuron function. The assays were conducted using flies of 2 different ages, 10 days old and 27 days old. At 10 days of age, a comparable age to that which the axonal area and volume were determined, the glial *Pex5* KD flies were trending downward in climbing ability compared to the control flies (Fig. 2d). At 27 days of age, the climbing ability of the glial *Pex5* KD flies was significantly impaired compared to the 2 control lines (Fig. 2d). These data indicate that the axonal swelling phenotypes observed in the glial *Pex5* KD flies are associated with motor defects, and these phenotypes worsen with age.

**Fig. 3.**
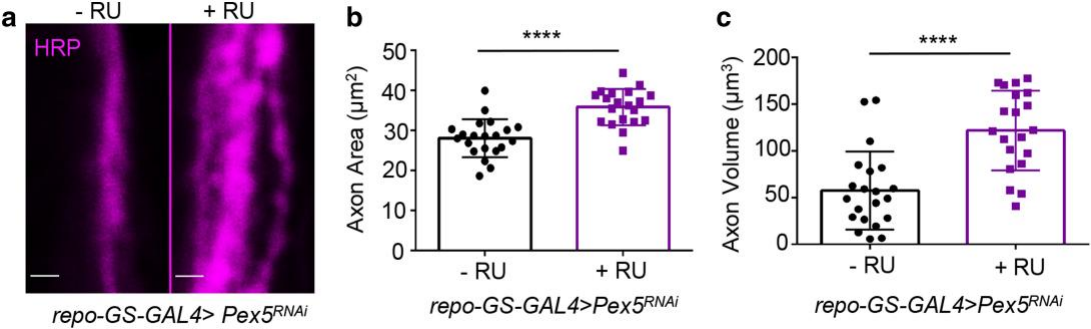
Adult-onset glia-specific KD of *Pex5* induces axonal swelling. a) Representative images showing axon morphology in wild-type (−RU, the left panel) and adult-onset glia-specific *Pex5* KD flies (+RU, the right panel). Axons are labeled with anti-HRP antibodies. The *repo-GS-GAL4* driver is used to knock down *Pex5* in adult glial cells. Mifepristone (RU486) is used as the chemical inducer. Scale bar: 1 μm. b, c) Quantification of axonal area and volume in flies with adult-onset glia-specific *Pex5* KD. *N* = 20, Student's *t*-test. *P < 0.05, **P < 0.01, **P < 0.001, and ****P < 0.0001.

To confirm the axonal defects are not due to any developmental issues, we employed a gene-switch system (*repo-GS-GAL4*) to knock down *Pex5* at the adult stages. Mifepristone (RU486) was added to fly food to active RNAi at 7 days of age, and axonal phenotypes were examined 7 days post-RU486 feeding. Similar to the results using constitutive *repo-GAL4*, adult-onset *Pex5* KD in glia cells significantly increased axonal area and volume (Fig. 3a–c). Together, these findings indicate that glial peroxisomes play an important role in supporting axonal structure and function in adult NMJ.

### KD of *Pex5* in specific glial subtypes induces axonal swelling

**Fig. 4.**
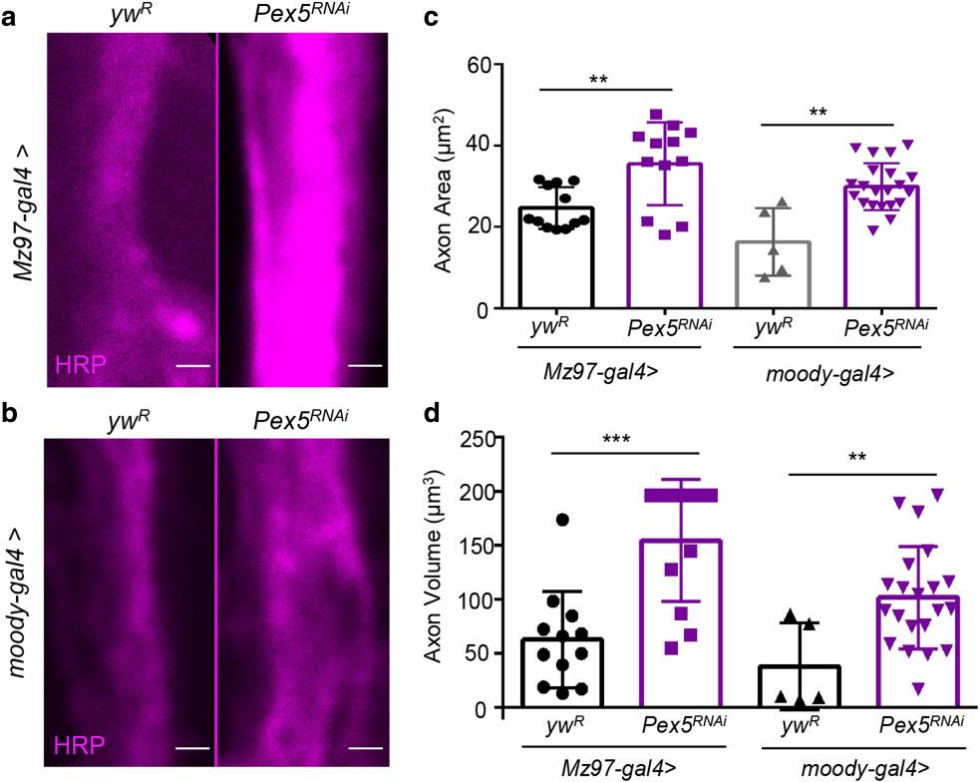
KD of *Pex5* in specific glial subtypes induces axonal swelling. a) Representative images showing axon morphology in wild-type (the left panel) and wrapping glia-specific *Pex5* KD (the right panel). Axons are labeled with anti-HRP antibodies. b) Representative images showing axon morphology in subperineurial/perineurial glia-specific KD of *Pex5*. Scale bar: 1 μm. c, d) Quantification of axonal area and volume in flies with wrapping glia- or subperineurial/perineurial glia-specific *Pex5* KD. *N* = 5–20, 1-way ANOVA. *P < 0.05, **P < 0.01, **P < 0.001, and ****P < 0.0001.

To further narrow down the glial subtypes responsible for the axonal swelling phenotype, we knocked down *Pex5* using specific glial cell drivers, *moody-GAL4* (subperineurial and perineurial glia driver) and *Mz97-GAL4* (wrapping glia driver) (Hartenstein 2011). Three major glial subtypes ensheathe the axon of fly NMJ. The wrapping glial cells are immediately outside the axon, whereas the subperineurial glial cells are outside the wrapping glia, and the peritoneal glia form the outermost cell layer (Hartenstein 2011). Interestingly, KD of *Pex5* in both wrapping glia and subperineurial/perineurial glia phenocopied the axonal swelling seen in the pan-glial *Pex5* KD. The axonal area and volume of glial subtype-specific KD of *Pex5* were larger than the control flies (Fig. 4a and d). Thus, these data indicate that Pex5 functions in multiple glial subtypes to regulate axonal structure and morphology.

### Impairment of VLCFA beta-oxidation and ether lipid biosynthesis in glial cells phenocopies *Pex5* KD and induces axonal swelling

**Fig. 5.**
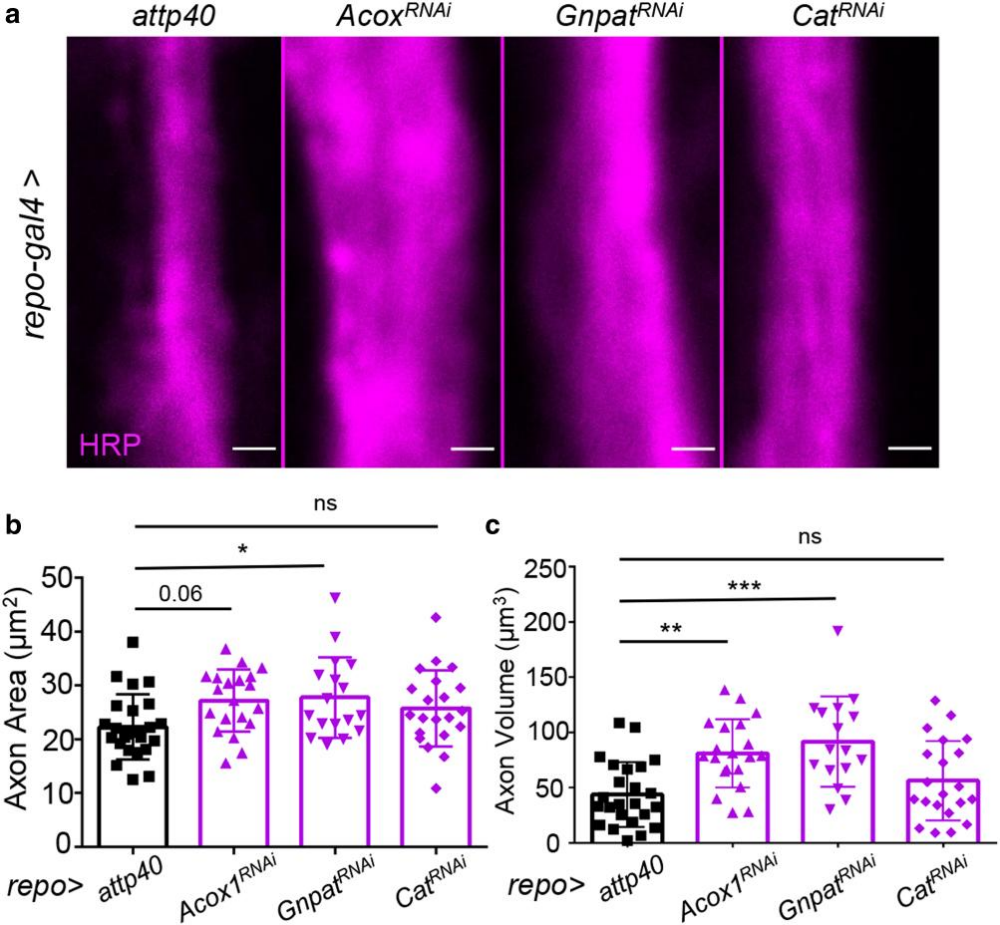
Impairment of VLCFA beta-oxidation and ether lipid biosynthesis in glial cells induces axonal swelling. a) Representative images showing axon morphology in *attp40* control (the left panel) and glia-specific KD of peroxisome matrix enzymes, *Acox1*, *Gnpat*, and *Cat*. Axons are labeled with anti-HRP antibodies. Scale bar: 1 μm. b, c) Quantification of axonal area and volume in flies with glia-specific KD of *Acox1*, *Gnpat*, and *Cat*. *N* = 15–20, 1-way ANOVA. *P < 0.05, **P < 0.01, **P < 0.001, and ****P < 0.0001.

Impairment of peroxisomal protein import via *Pex5* KD could alter various peroxisome functions, such as ROS metabolism, beta-oxidation of VLCFAs, and biosynthesis of ether phospholipids. Next, we conducted a genetic analysis to determine which peroxisomal function is involved in glia–neuron communication. We knocked down 3 peroxisomal enzymes using *repo-GAL4* driver. These peroxisomal enzymes are Acox1 (Acyl-CoA oxidase 1 involved in VLCFA beta-oxidation), Gnpat (glyceronephosphate O-acyltransferase involved in ether lipid biosynthesis), and Cat (catalase for hydrogen peroxide detoxification). As shown in Fig. 5, glial-specific KD of *Acox1* significantly induced axonal volume, while glial-specific KD of *Gnpat* significantly induced both axonal area and volume (Fig. 5a–c). In contrast, glial-specific KD of *Cat* did not affect axonal morphology (Fig. 5a–c), despite a significant KD of *Cat* mRNA (Supplementary Fig. 1). The *catalase* KD results were unexpected, as elevated ROS production is often associated with neuroinflammation (Chung *et al*. 2020; Simpson and Oliver 2020). Together, these findings reveal specific peroxisomal functions, such as ether lipid biosynthesis, in maintaining axonal structure and integrity.

### Defective peroxisomal import upregulates pro-inflammatory cytokine upd3 in glia, while motor neuron-specific activation JAK-STAT signaling increases axonal swelling

**Fig. 6.**
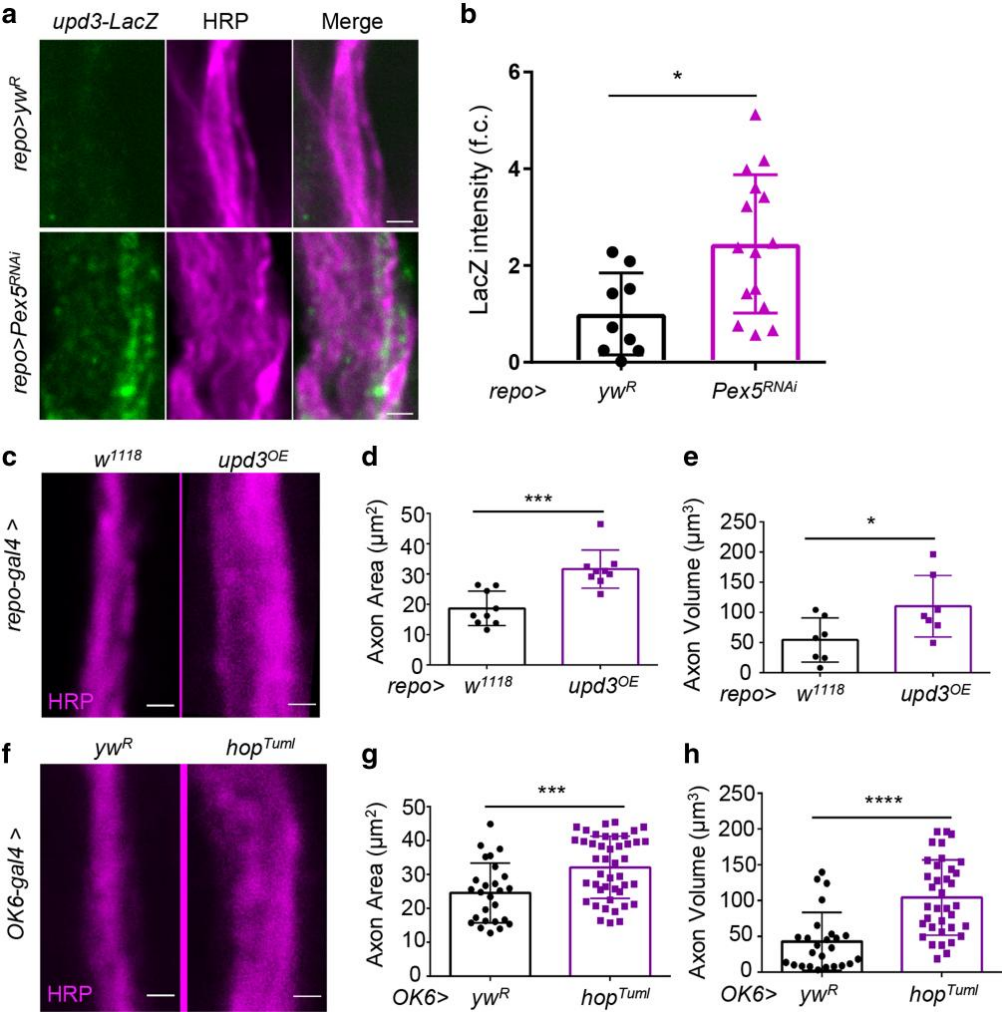
Defective peroxisomal import upregulates pro-inflammatory cytokine upd3 in glia, and motor neuron-specific activation JAK-STAT signaling increases axonal swelling. a) Representative images showing *upd3-LacZ* activity in wild-type (the top row) and glia-specific KD of *Pex5* (the bottom row). Axons are labeled with anti-HRP antibodies. Scale bar: 1 μm. b) Quantification of LacZ intensity between control and glial *Pex5* KD. *N* = 10–15, unpaired 2-tailed Student's *t*-test. c) Representative images showing axon morphology in wild-type (the left panel) and glia-specific overexpression of *upd3* (the right panel). Axons are labeled with anti-HRP antibodies. Scale bar: 1 μm. d, e) Quantification of axonal area and volume in flies with glia-specific *upd3* overexpression. *N* = 6–9, Student's *t*-test. f) Representative images showing axon morphology in wild-type (the left panel) and motor neuron-specific expression of *hop*^*Tuml*^ (the right panel). Axons are labeled with anti-HRP antibodies. Scale bar: 1 μm. g, h) Quantification of axonal area and volume in flies with motor neuron-specific *hop*^*Tuml*^ expression. *N* = 20–30, Student's *t*-test. *P < 0.05, **P < 0.01, **P < 0.001, and ****P < 0.0001.

We previously reported that impaired peroxisomal import increased the production of pro-inflammatory cytokine upd3, a 4-helix bundle interleukin-6 (IL-6) type pro-inflammatory cytokine, which activates JAK-STAT signaling in *Drosophila* (Huang *et al*. 2020). We wondered whether *Pex5* KD could also upregulate *upd3* in glial cells, which in turn induced axonal inflammation and swelling. To test this idea, we first examined the expression of *upd3* using a reporter line (*upd3-LacZ*) upon glial *Pex5* KD. Consistent with our prediction, glia-specific KD of *Pex5* resulted in a 2-fold induction of *upd3-LacZ* expression (Fig. 6a and b). We further showed that overexpression of *upd3* in glial cells increased axon area (1.7-fold) and volume (2-fold) (Fig. 6c–e). The pro-inflammatory cytokine upd3 is known to systemically upregulate the JAK-STAT pathway in response to tissue injuries, excess dietary lipid, and other stress conditions (Pastor-Pareja *et al*. 2008; Buchon *et al*. 2009; Woodcock *et al*. 2015). We then asked whether activation of JAK-STAT signaling in neuronal tissue phenocopies glial overexpression of *upd3*. To test this, we used a motor neuron driver (*OK6-GAL4*) to overexpress *hop*^*Tuml*^, a dominant hyperactive form of the fly JAK kinase that results in constitutive activation of the JAK-STAT pathway (Silver and Montell 2001). As expected, motor neuron-specific overexpression of *hop*^*Tuml*^ caused a significant increase in axon area (1.4-fold) and volume (2.1-fold) (Fig. 6f–h). Taken together, our findings indicate that impaired peroxisomal import upregulates the production of pro-inflammatory cytokine upd3, which signals to motor neurons and alters axonal structure.

## Discussion

**Fig. 7.**
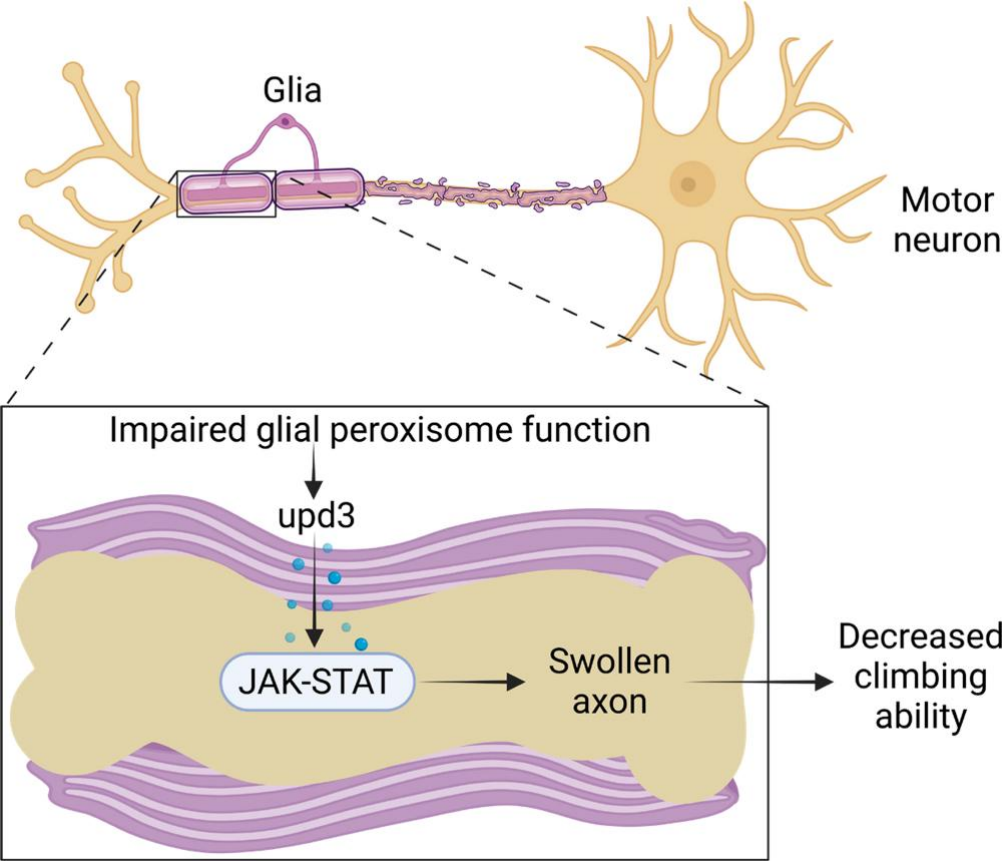
The proposed model shows that impaired glial peroxisomal import function induces the production of pro-inflammatory cytokine upd3, which nonautonomously activates JAK-STAT in motor neurons and causes axonal swollen and locomotion defects.

Despite its important role in neuronal development and neurodegeneration, how peroxisomes regulate glia–neuron communication remains largely unknown. Using a *Drosophila* NMJ model, we discovered that glia-specific KD of peroxisome import receptor *Pex5* significantly induces locomotion defects and axon swelling of the motor neurons. The axon swelling is likely caused by glia-derived pro-inflammatory cytokine upd3 that activates JAK-STAT signaling in motor neurons. We further show that peroxisomal beta-oxidation and ether lipid biosynthesis are involved in glia–neuron communication. Our findings reveal an important role of peroxisome in glia–neuron communication and JAK-STAT-mediated neuroinflammation (Fig. 7).

Most of the previous studies focus on the cell-autonomous role of peroxisome in neuronal tissues, rather than glial cells. Additionally, how glial peroxisomes protect neuronal health is largely unknown. Our observations on axon swelling and neuroinflammation in glial *Pex5* KD flies are similar to previous studies in oligodendrocyte-specific *Pex5* KO mice (Kassmann *et al*. 2007). However, in this study, the underlying mechanisms for neuroinflammation and the secretory factors mediating glia–neuron communication are not identified. The present study uncovered pro-inflammatory cytokine upd3, the homolog of mammalian IL-6, as the secretory factor downstream of glial peroxisome to induce axon defects. It is likely there are other glia-derived secretory factors that signal to neurons to regulate neuroinflammation and neuronal homeostasis. Our model system offers a unique opportunity for the genetic dissection of signaling molecules mediating glia–neuron communication, especially under peroxisome dysregulation.

The JAK-STAT pathway is well known for its role in innate immunity and inflammation from flies to mammals (Agaisse and Perrimon 2004; Hu *et al*. 2021; Sarapultsev *et al*. 2023). Indeed, abnormal JAK-STAT activation has been linked to autoimmune diseases (such as systemic lupus erythematosus and rheumatoid arthritis), various cancers, and neuroinflammatory-associated diseases (such as multiple sclerosis and Parkinson's disease) (Hu *et al*. 2021; Sarapultsev *et al*. 2023). JAK-STAT pathway plays an essential role in the transcriptional activation of immune-related genes, including the expression of more than 30 cytokines (Agaisse and Perrimon 2004; Hu *et al*. 2021). JAK-STAT activation has been shown to contribute to inflammation as a pathogen response (Yan *et al*. 2018). In *Drosophila*, the JAK-STAT signaling pathway has been shown to increase immune-related genes and cause differential immune cell differentiation (Myllymäki and Rämet 2014). The role of JAK-STAT in neuroinflammation has been unveiled in many previous studies of neurodegenerative diseases. Recent studies show that α-synuclein can activate the JAK-STAT pathway in microglia and macrophages of a model of Parkinson's disease, while the JAK1/2 inhibitor, AZD1480, attenuates the degeneration of dopaminergic neurons (Qin *et al*. 2016). We previously showed that JAK-STAT signaling is activated in aged fly hearts in response to age-related peroxisome import impairment and the induction of hepatic cytokine *upd3* expression (Huang *et al*. 2020). Given the role of JAK-STAT in neuroinflammation, it is not a total surprise to find that glia-specific KD of *Pex5* activates JAK-STAT and alters axon structure. Collectively, the findings from us and others support a vital role of the peroxisome-JAK-STAT axis in neuroinflammation and neurodegenerative diseases.

In the present study, we show that glia-specific KD of 2 peroxisomal enzymes, Acox1 and Gnpat, induces axon defects. These results indicate an essential role of glial VLCFA beta-oxidation and ether lipid biosynthesis in neuroinflammation. It is previously known that peroxisomal dysfunction can lead to altered neuronal function and perturbation of lipid metabolism in *Drosophila* (Mast *et al*. 2011). Patients with ACOX1 deficiency typically have an abnormal accumulation of VLCFAs and exhibit seizures, motor defects, and hearing and visual loss (Ferdinandusse *et al*. 2007). In *Drosophila*, *dACOX1* null mutants also exhibit severe neurodegeneration of the retina and vision loss (Chung *et al*. 2020). However, little is known about the role of glial beta-oxidation of VLCFA in neuroprotection and neuroinflammation. It is likely that loss of *ACOX1* promotes lipid peroxidation and ROS production in glial cells, which disrupts neuronal function via secretory cytokines and other intercellular communication factors.

Among all peroxisome functions, ether lipid biosynthesis is one of the understudied, yet essential processes that play critical roles in organelle membrane dynamics, neuronal protection, and antioxidation (Fig. 3a). Ether phospholipids (mainly plasmalogens) comprise about 18–20% of the total phospholipid mass in animals and humans and are the major components of the cell membrane, especially mitochondrial inner and outer membranes (Honsho *et al*. 2008; Park *et al*. 2019). Plasmalogens are about 80% of ethanolamine glycerophospholipids in the myelin sheath of the CNS (Dean and Lodhi 2018). Plasmalogen deficiency has been linked to impaired mitochondrial dynamics, respiratory disorders, cardiomyopathy, and Alzheimer's disease (Braverman and Moser 2012; Kimura *et al*. 2019; Dorninger *et al*. 2020). However, the role of ether lipids in glial function and glia–neuron communication is largely unknown. The findings in the present study suggest that glial ether lipid biosynthesis is crucial for neuronal health, especially axonal integrity. It is worth mentioning that among all 3 peroxisomal functions examined, the defects in ether lipid biosynthesis cause the most significant axonal swelling phenotype. Given that ether phospholipids are essential components of cell membranes, we speculate that glial cells might produce and supply ether phospholipids to support axonal structure. Loss of ether phospholipid biosynthesis in glial cells can result in impairment of axonal membrane structure and induction of neuroinflammation.

In summary, our findings reveal an essential role of glial peroxisomes in neuroprotection and axonal integrity. In particular, loss of glial ether lipid biosynthesis is responsible for the elevated neuroinflammation. We further identify JAK-STAT pathway as the downstream signaling mediating peroxisome-regulated neuroinflammation. The elucidation of the role of peroxisome in neuroinflammation and glia–neuron communication not only provides valuable insights into our understanding of neuroinflammation diseases but also the mechanisms underlying peroxisomal biogenesis disorders in humans.

## Supplementary Material

jkae243_Supplementary_Data

## Data Availability

The authors affirm that all data necessary for confirming the conclusions of the article are present within the article and figures. All of the *Drosophila* stocks are obtained from Bloomington Stock Center or individual labs; see identifiers and sources listed in the Materials and Methods section. Supplemental material available at G3 online.
