# Supplementary material for: Glial peroxisome dysfunction induces axonal swelling and neuroinflammation in Drosophila
Source: G3 (Bethesda). 2024 Oct 10;15(1):jkae243. doi: 10.1093/g3journal/jkae243 (PMC11708211; doi:10.1093/g3journal/jkae243)
Supplement: jkae243_Supplementary_Data [file jkae243_supplementary_data.pdf]

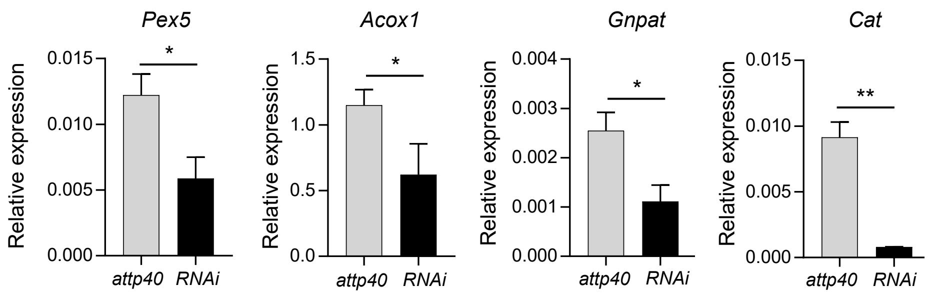

Figure S1. qRT-PCR analysis for validation of the knockdown efficiency of RNAi lines for *Pex5, Acox1, Gnpat,* and *Cat*. N=3, Student's t-test. *: p<0.05, **: p<0.01.
